# 3D In Vivo Models for Translational Research on Pancreatic Cancer: The Chorioallantoic Membrane (CAM) Model

**DOI:** 10.3390/cancers14153733

**Published:** 2022-07-31

**Authors:** Eric Pion, Julia Karnosky, Sofie Boscheck, Benedikt J. Wagner, Katharina M. Schmidt, Stefan M. Brunner, Hans J. Schlitt, Thiha Aung, Christina Hackl, Silke Haerteis

**Affiliations:** 1 Institute for Molecular and Cellular Anatomy, University of Regensburg, 93053 Regensburg, Germany; eric.pion@stud.uni-regensburg.de (E.P.); sofie.boscheck@stud.uni-regensburg.de (S.B.); thiha.aung@th-deg.de (T.A.); 2 Department of Surgery, University Hospital Regensburg, 93053 Regensburg, Germany; julia.kanovsky@ukr.de (J.K.); benedikt.wagner@ukr.de (B.J.W.); katharina.schmidt@ukr.de (K.M.S.); stefan.brunner@ukr.de (S.M.B.); hans.schlitt@ukr.de (H.J.S.); christina.hackl@ukr.de (C.H.); 3 Faculty of Applied Healthcare Science, Deggendorf Institute of Technology, 94469 Deggendorf, Germany

**Keywords:** pancreatic cancer, pancreatic ductal adenocarcinoma (PDAC), chorioallantoic membrane (CAM) model, 3D in vivo model

## Abstract

**Simple Summary:**

The 5-year overall survival rate for all stages of pancreatic cancer is relatively low at about only 6%. As a result of this exceedingly poor prognosis, new research models are necessary to investigate this highly malignant cancer. One model that has been used extensively for a vast variety of different cancers is the chorioallantoic membrane (CAM) model. It is based on an exceptionally vascularized membrane that develops within fertilized chicken eggs and can be used for the grafting and analysis of tumor tissue. The aim of the study was to summarize already existing works on pancreatic ductal adenocarcinoma (PDAC) and the CAM model. The results were subdivided into different categories that include drug testing, angiogenesis, personalized medicine, modifications of the model, and further developments to help improve the unfavorable prognosis of this disease.

**Abstract:**

Pancreatic ductal adenocarcinoma (PDAC) is a highly aggressive cancer with adverse outcomes that have barely improved over the last decade. About half of all patients present with metastasis at the time of diagnosis, and the 5-year overall survival rate across all stages is only 6%. Innovative in vivo research models are necessary to combat this cancer and to discover novel treatment strategies. The chorioallantoic membrane (CAM) model represents one 3D in vivo methodology that has been used in a large number of studies on different cancer types for over a century. This model is based on a membrane formed within fertilized chicken eggs that contain a dense network of blood vessels. Because of its high cost-efficiency, simplicity, and versatility, the CAM model appears to be a highly valuable research tool in the pursuit of gaining more in-depth insights into PDAC. A summary of the current literature on the usage of the CAM model for the investigation of PDAC was conducted and subdivided into angiogenesis, drug testing, modifications, personalized medicine, and further developments. On this comprehensive basis, further research should be conducted on PDAC in order to improve the abysmal prognosis of this malignant disease.

## 1. Introduction

### 1.1. Pancreatic Cancer

#### 1.1.1. Epidemiology

In 2018, 9860 men and 9160 women in Germany were diagnosed with pancreatic cancer and despite enormous research efforts, it remains one of the cancer types with the lowest 5-year survival, second only to mesothelioma [1,2]. Thus, there is only a minor difference between incidence and prevalence. It is the fourth leading cause of cancer-related death in Germany, as well as in the USA, causing approximately 270,000 deaths annually worldwide [1,2]. Approximately 95% of all pancreatic neoplasms are adenocarcinomas, which develop from a malignant transformation of the exocrine part of the pancreas. The average age at diagnosis is between 60 and 80 years. About 90% of pancreatic cancers occur sporadically while roughly 10% are hereditary. There is no significant difference in the yearly incidence between male and female patients [1,3].

Upon the initial diagnosis of PDAC, about 50–55% of patients have already developed distant metastases, 20–25% of patients have a locally advanced tumor stage, and merely 20% have a potentially resectable tumor. Long-term survival is an exception and it is strongly linked to early diagnosis. The published five-year survival rate across all tumor stages is only 6% but ranges from 2–9% worldwide [4]. Currently, the most important factor in determining the outcome for the patient is the disease stage at the initial diagnosis [5].

#### 1.1.2. Risk Factors

Some diseases that appear to be associated with a higher risk of developing PDAC include chronic pancreatitis, diabetes mellitus type 2, and syndromes such as Peutz-Jeghers- or Li-Fraumeni-Syndrome. Hereditary predispositions have also been identified through germline mutations in BRCA2, PALB2, CDKN2A, STK11, PRSS1 genes [6]. Additional risk factors include smoking, age, and dietary habits such as high fat, high meat, low vegetable, and folate intake. Helicobacter pylori infections are also potentially linked to the development of PDAC. However, the etiology is still unclear in most cases.

#### 1.1.3. Diagnosis and Staging

This type of cancer is generally diagnosed at a more advanced stage because a small tumor within the pancreas does not cause pain or functional impairment [5]. Symptoms at presentation may include persistent epigastric pain that can radiate to the back, painless jaundice (Courvoisier sign) which occurs in advanced tumor stages, and only if the neoplasm is located within the head of the pancreas as well as lack of appetite, weight loss, and diarrhea [6].

Currently, there are a variety of imaging modalities available for the detection of a suspected pancreatic neoplasm. Some are non-invasive like ultrasound, CT scan, and MRI, while others, such as MRCP, ERCP, and endoscopic ultrasound are invasive [7]. If a solid mass is visible within the pancreas, the tumor marker CA 19-9 can also be determined to provide additional information. Unfortunately, this test only has a sensitivity and specificity of approximately 80%, and an even lower sensitivity for small PDACs, therefore it does not appear to be a satisfactory screening tool [8].

Diagnosis is followed by clinical staging, which incorporates the TNM and UICC classification. Furthermore, infiltration/affection of the major local blood vessels has to be evaluated. The primary resection is the only treatment that has a curative intention for patients with non-metastasized, potentially resectable PDAC [9]. In these cases, the published five-year survival rates are 25–30%. The potential resectability of these tumors mainly depends on their invasion of major blood vessels within the anatomical region. This is even more important for cases in which the tumor is located within the head of the pancreas because several large arteries (celiac axis, hepatic artery, and the superior mesenteric artery) should be free of tumor to perform a successful resection. However, the International Association of Pancreatology (IAP) introduced the ABC criteria that determine the resectability, not only on the localization regarding the blood vessels but also by incorporating the ECOG status, the lymph node status, as well as tumor markers. This underlines the complexity of determining valid protocols for the resectability of PDAC [3,10,11].

#### 1.1.4. Surgery

When performing surgery on patients with PDAC, venous and arterial resections result in higher postoperative morbidity, mortality, and impaired long-term survival. Some criteria for resection also vary among surgeons and associations [12]. If no contact with the celiac axis, hepatic artery, or the superior mesenteric artery is found, a tumor is classified as resectable, whereas tumors with infiltration of the superior mesenteric vein, major mesenteric, or celiac vessels of more than 180° are considered unresectable. However, the portal vein can be resected if it is tumor encased. If the tumor is in an advanced stage at diagnosis (local irresectability or distant metastases), a histological sample must be obtained to start a chemotherapy regimen as part of palliative treatment. In the past, these tumors were all determined to be unresectable while today there is a possibility to reevaluate the resectability in specific cases within research protocols after neoadjuvant chemotherapy. Different classifications are used in the literature, some publications call these tumors “borderline resectable” [13,14].

#### 1.1.5. Adjuvant, Neoadjuvant, and Palliative Therapy

Chemotherapy is the second column of the treatment of PDAC in all stages. It can be classified into three major treatment options: adjuvant therapy, palliative therapy in unresectable/metastatic patients, and neoadjuvant/induction treatment (mainly in borderline resectable patients and clinical trials) [15]. After surgical resection, patients should always receive adjuvant chemotherapy. There are different approved chemotherapy protocols for this purpose. The most common regimens that are currently used are FOLFIRINOX and Gemcitabine-based protocols [12]. Preoperative therapy of resectable or metastatic PDAC is considered to be neoadjuvant treatment. Neoadjuvant therapy is the focus of many ongoing studies, as it is generally better tolerated than adjuvant therapy and may decrease the risk of complications during or after surgery [16]. It can be considered a systemic therapy, as it targets distant micro metastases and circulating cancer cells, while at the same time downsizing the primary tumor, which results in a higher rate of R0-resection [12]. Advanced stages are treated with palliative chemotherapy if the general condition of the patient is adequate. Targeted therapies using monoclonal antibodies have been very successful in the treatment of various cancers such as colorectal cancer or breast cancer, but have failed in regard to PDAC with the EGFR inhibitor erlotinib representing a limited exception [17,18]. In many different types of cancers, the development of new immunotherapies and individualized tumor therapies have led to a strong improvement in therapeutic outcomes. Regarding PDAC, various trials are currently investigating potential therapeutic options that include radiofrequency ablation [19], microwave ablation, [20] and local anti-KRAS therapy [21] among others. Unfortunately, these have not been successful in the treatment of patients with PDAC [22].

#### 1.1.6. Cell Culture Models

Traditional (2D) cell culture models using mouse or human cell lines have been the basis for a large part of the research that has been conducted on PDAC and other cancers. While being relatively cost and time-efficient these basic research models facilitated the discovery of genes, oncogenic pathways, and key processes like cell migration [23]. Regarding PDAC, difficulties in accessing patient-derived tissue have led to the use of a limited number of cell lines in preclinical cell culture models which carries the risk of genetic and phenotypic drift of the cells [24]. Despite carrying different genetic mutations, most cultured PDAC cell lines depict similar morphological features and can be categorized into three subtypes (classical, quasi-mesenchymal, and exocrine-like) [25]. These subgroups are based on differences regarding epithelial or mesenchymal-related genes but have been questioned recently due to potential contamination of the exocrine-like subtype [26]. This highlights the potential hazards of studies that are exclusively based on the use of cell lines. Nevertheless, PDAC is a disease that proliferates within a tumor microenvironment and new 3D models are necessary that can mimic this environment that includes fibroblasts, cells of the immune system next to others, in order to gain new insights [25,27,28].

#### 1.1.7. Preclinical Models

To improve the treatment of PDAC, it is necessary to gain a better understanding of the biological and molecular mechanisms that influence tumor growth and malignancy. For this purpose, additional 3D-in-vivo tumor models appear indispensable. In recent years, improvements regarding patient-derived cancer transplant models have become the main focus of research for PDAC, since they are supposed to display more realistic responses to potential therapies [29]. Durymanov et al. describe the subcutaneous implantation of 3D pancreatic adenocarcinoma spheroids into mouse models in order to mimic a more accurate extracellular matrix of the human tumor microenvironment in comparison to tumor cell suspensions [30]. Another application involves patient-derived pancreatic ductal adenocarcinoma cells that were grown in 3D culture chambers prior to injection into mouse models with the aim of establishing a drug screening model [31]. A short overview of currently used mouse models is provided in Table 1.

**Figure 1:**
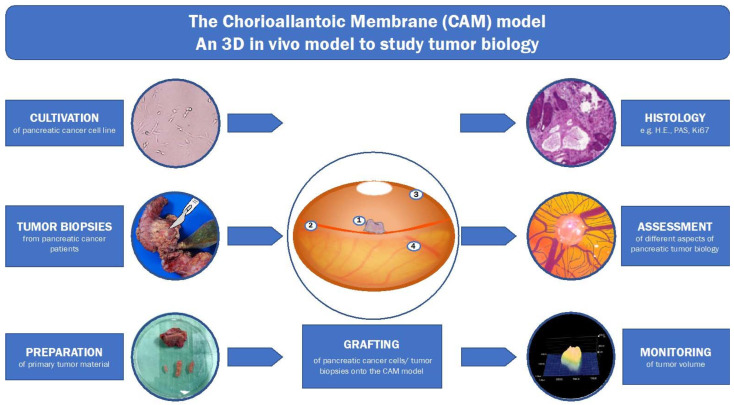
Illustration of the 3D in-vivo model for the study of pancreatic ductal adenocarcinoma (1: tumor, 2: CAM-vessels, 3: eggshell, 4: CAM). Cultivation of pancreatic cancer cell lines, as well as tumor biopsies of pancreatic ductal adenocarcinoma patients, can be prepared and grafted onto the CAM model. After a certain cultivation time with the possibility to test potential therapeutics one can assess different aspects of the tumor development. These aspects include histology, tumor angiogenesis, and tumor volume among many other possibly interesting readouts of tumor development.

One in vivo model that has recently gained a lot of attention as a platform for the assessment of tumor angiogenesis, [32] and as a drug testing platform [33,34], is the chorioallantoic membrane (CAM) model (see Figure 1). Furthermore, it has been used for the investigation of multiple forms of cancer and has proven its high level of versatility [35,36,37,38]. Because of its simple protocol, cost efficiency, and growth-promoting environment, it has also been utilized as a drug testing platform for primary pancreatic ductal adenocarcinoma cells. In a recent study, Rovithi et al. investigated the effects of combination chemotherapy of gemcitabine and crizotinib on immunohistochemical and genetic analyses which included microRNA profiling [39]. This example underlines the utility of the CAM model as an in vivo model that incorporates a vast array of molecular, biological, histopathological, and pharmacological research opportunities.

**Table 1:** Overview of current murine models for the investigation of pancreatic cancer.

Study	Model	Subtype	Advantages	Disadvantages	Treatment
[40]	Cell line-derived xenograft models	Orthotopic xenografts	More realistic tumor biology and environment (than heterotopic models) Imitation of human pathologies (e.g., obstructive jaundice, organ invasion) possible	Challenging injection Time-consuming and relatively costly Tumor formation uncertain	2 weeks, or 3 weeks
[41]		Heterotopic xenografts (mostly subcutaneous (s.c.) injection in the flank)	Tumor growth macroscopically observable Promising drug testing platform Higher reproducibility	Less realistic tumor biology and environment (than orthotopic models) Metastasis less likely Different blood supply	4 weeks, or up to 47 days
[42]	Syngeneic xenograft models	Orthotopic xenografts	Immunocompetence enables the study of the immune response Tumor metastasis more likely Accelerated disease progression	No human cells, limited relevance Lack of oncogenic mutations High variation of results due to different protocols	Up to 27 days
[43]		Heterotopic Xenografts (mostly s.c.)	Tumor easily accessible for measurements Immunocompetence enables the study of the immune response High reproducibility	No human cells, limited relevance Metastasis unlikely Lack of oncogenic mutations (unlike GEMMs)	52 days, or 2 months
[44]	Xenogeneic xenograft models	Orthotopic xenografts	Personalized medicine Higher clinical relevance (than heterotopic models) Higher reproducibility due to uniform growth patterns	Costly and labor-intensive Immunocompromised mice tumor sutured onto the pancreas instead of intrapancreatic growth	8 weeks
[45]		Heterotopic Xenografts (mostly s.c.)	Tumor growth macroscopically observable and measurable Human tumor cells Higher reproducibility	Immunocompromised Low metastasis rates No realistic infiltration into neighboring organs	20 days of drug testing
[46]	Chemically induced-xenograft models		High clinical relevance Drug screening platform Hamster’s pancreas is more similar to human pancreas	Lack of reproducibility Inconsistency Mostly performed in hamsters which are more expensive	24 weeks
[47]	Patient derived-xenograft models	Orthotopic xenografts	Comparison with patient survival possible Individualized therapy testing Gene expression largely preserved	High costs and workload Human cells are replaced by murine cells over time High costs and large data sets difficult to acquire	Up to 46 months
[48]		Heterotopic xenografts (mostly s.c.)	Human tumor cells and stroma Tumor growth macroscopically observable Genotype preserved during early stages Promising drug testing platform Personalized medicine	High costs and workload Human cells are replaced by murine cells over time Tumor vessels and microenvironment differ from origin	28 days
[49]	Genetically engineered murine models (GEMMs)		Analysis of specific oncogenes Immunocompetence High-quality preclinical drug testing platform Multistep progression of cancer observable	High costs Prolonged tumorigenesis No exact mimicry of complex genetic alterations of human tumors	100 days

### 1.2. 3D-In-Vivo-Tumor-Model

The CAM is formed by the synthesis of the chorion and the allantois during the embryogenesis of a chicken and resembles a highly vascularized extra-embryonic membrane within chicken eggs [50,51]. It is attached to the inner part of the eggshell and mainly functions as a membrane for the gas exchange of the developing embryo [52]. It also enables the transportation of electrolytes and participates in osteogenesis by mobilizing calcium from the eggshell to initiate the process of bone mineralization [53].

Because of the easy accessibility of the CAM and its high level of vascularization, the main focus of initial research projects was the in vivo investigation of angiogenesis and antiangiogenic drugs [54]. Today, the CAM model is used in many different fields such as bioengineering, transplant biology, cancer research, and drug development [52].

Currently, there are many different protocols in place that describe a successful implementation of the CAM model. To access the CAM, three different approaches can be found in the literature: the dropped membrane technique (in ovo), the Zwilling technique (in ovo) and the shell-less technique (ex ovo) [55]. While all techniques have certain advantages and disadvantages, the dropped membrane technique is the most commonly used among the three. The eggs are usually placed in a special incubator which assures constant humidity and temperature for the development of the CAM during the experiment. In the incubator, the eggs are positioned on a rotating device to prevent the embryo from attaching to one side of the eggshell until access to the CAM is enabled. After opening the eggshell, it is essential to keep the eggs as clean as possible and to seal the opening whenever possible to prevent contamination. There is no universally accepted protocol that provides instructions on which day to engraft or harvest tumors from the CAM. The exact methodology depends on the type of the experiment and must be evaluated for each study in advance.

In the last decades, many different tumor cells and primary tumor samples have been successfully grafted onto the CAM. These CAM tumors have not only grown in size on the CAM, but also induced angiogenesis, invaded the CAM, and even metastasized. Therefore, the CAM model can be regarded as an in vivo model that incorporates the 3R principle (“replace, reduce, refine”) for the prevention of unnecessary suffering of animals due to research purposes.

Here, we aim to provide a comprehensive summary of the current findings and methodologies of the utilization of the CAM model for the investigation of pancreatic cancer.

## 2. Materials and Methods

A literature search in PubMed using the concepts “pancreatic cancer” and “chorioallantoic membrane” was conducted. The search was last updated on 18 December 2021. Both thesauri and a broad range of synonyms were used. We limited the study to publications in English. Screening was done using the full-text version of the publications. We did not limit the search for a study type, publication type, or publication date. The reference lists of the included studies were also screened for additional studies. All records were imported into EndNote software and the full-text version was obtained for all of them. All studies in which PDAC cells were grafted onto the chorioallantoic membrane were included.

## 3. Results

### 3.1. Evidence Search

**Figure 2:**
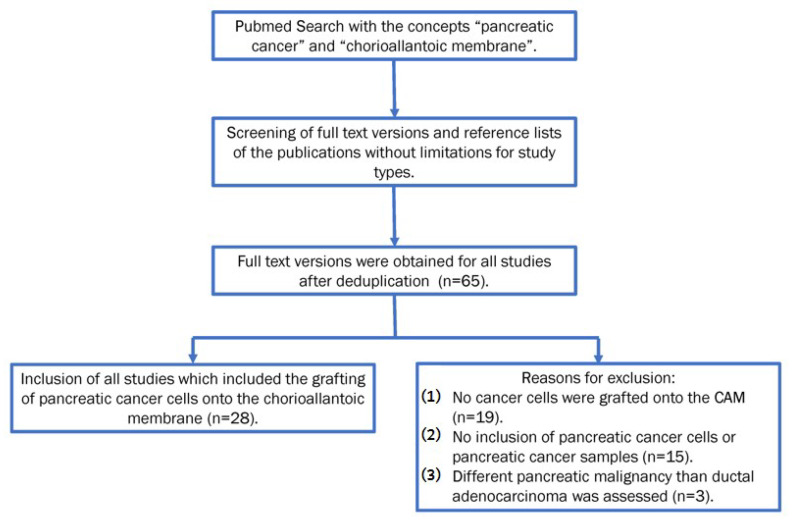
Flowchart illustrating the selection of in- or exclusion of studies.

The initial PubMed search yielded 76 results. After deduplication, 67 results were evaluated for inclusion. Two of the results were excluded because they were published in Chinese [56,57]. For the remaining 65 publications, the full-text versions were obtained. Of these 65 publications, 28 were included in this review because they met the inclusion criteria. The most prevalent reason for exclusion was that no cancer cells were grafted onto the CAM (*n* = 19). The second most common reason was that no pancreatic cancer cells or samples were included (*n* = 15). Additonally in a few cases, a different kind of pancreatic neoplasm was analyzed, for example, IMPN or neuroendocrine tumor of the pancreas (*n* = 3) (see Figure 2).

We divided the included studies into the following subgroups depending on the focus of the described research. These subgroups include angiogenesis, (publications of Table 2), drug testing (Table 3), modifications of cells engrafted onto the CAM and further developments, and personalized medicine: Endpoints and read-outs were Growth/proliferation rate, tumor weight and viability, angiogenesis and angioinvasion, invasion of the CAM and metastasis, histology and molecular biology.

### 3.2. Angiogenesis

The CAM Model was first developed for the study of angiogenesis, and while it is now used for a wide range of different purposes, it is still very frequently applied as an angiogenesis assay (see Table 2). A multitude of different protocols have been published for the assessment of angiogenesis in the past and mostly aim at testing potential therapeutics and their effects on the development of the CAM vessels.

Angioinvasion is a critical step for tumor growth and the dissemination of metastases. In 2009, Büchler et al. used the CAM model to analyze angioinvasion and the development of metastases for several different pancreatic cancer cell lines [58]. They tested the angioinvasive potential under both normoxia and hypoxia under the influence of urokinase-type plasminogen activator receptor (uPAR) antibodies. Angioinvasion was strongly increased by hypoxia, and the authors concluded that the angioinvasive potential of pancreatic cancer is highly dependent on uPAR expression.

Protein kinase D (PKD) 2 production is induced by hypoxia in pancreatic cancer cells. Azoitei et al. aimed to directly examine the influence of PKD on the vessel formation of the CAM [59]. After silencing chicken PKD 1 and PKD 3, they noticed a decreased tumor formation. A decrease in tumor growth by more than 80% was observed when human PKD 2 was depleted within the tumor cells. This also resulted in a decrease in the formation of chicken blood vessels.

Laklai et al. investigated the role of thrombospondin-1 (TSP-1) in tumor growth and angioinvasion, which is regulated by the activity of Somatostatin receptor subtype 2 (sst2) [60]. A strong decrease of tumor-induced angioinvasion into the CAM when sst2 was expressed in the implanted pancreatic cancer cells.

In 2016, Shen et al. reported the implanting of MiaPaCa-2 cells using gelatin sponges to observe the effect of Epidermal growth factor-like domain 7 (EGFL7) on tumor angiogenesis [61]. It was shown that silencing EGFL7 expression inhibited the effect on the formation of microvessels. They photographed the eggs on day 10 after incubation and counted the vessels entering the gelatin sponges within the focal plane of the CAM at a magnification of 50× to quantify the effects.

In a study published in 2019, Quan et al. used the CAM model as an angiogenesis assay to analyze, whether Ezrin promotes pancreatic cancer cell proliferation and invasion [62]. They counted the total number of vessel branching points after 24 and after 120 h of engraftment. The results showed that Ezrin could play a role in regulating angiogenesis in pancreatic cancers. In addition, 187 pancreatic cancer samples were analyzed and overexpression of Ezrin and YAP (Yes-associated protein) was observed and correlated with a poor prognosis.

### 3.3. Drug Testing

Since its development, the CAM model has been used as a drug testing platform not only for antiangiogenetic drugs but also for anticarcinogenic drugs (see Table 3). Because of the versatility of the model, a multitude of drugs can be applied locally onto CAM tumors or the surrounding area, intravascular or intratumoral injection is also possible. Effectiveness can then be assessed by volume, weight, and angiogenesis measurements next to histological and molecular analyses of the tumors.

In two studies, substances of a natural origin were tested on pancreatic cancer cells. Sudha et al. tested the antiangiogenic effect of pomegranate fruit extract on pancreatic cancer cell line tumors on the CAM [63]. In this study, tumor growth and tumor angiogenesis were determined on day 7 after implantation. Compared to the controls, the pomegranate extracts significantly reduced tumor weight and the number of small blood vessels. Additionally, tumor hemoglobin (Hb) was decreased and determined as a novel form of measurement for the angiogenesis of the tumor.

Mousa et al. tested the potential effects of nanoformulated bioactive compounds of several different natural substances on pancreatic cancer [64]. They used SUIT2-Luc cells, which express firefly luciferase activity. The effects on tumor weight and angiogenesis were determined and bioluminescence was used to detect viable tumor cells. Also, 3,3′-diindolylmethane (DIM) and Ellagic acid (EA) were tested in their natural as well as their nanoencapsulated form. A greater decrease in tumor weight, tumor viability and tumor angiogenesis was seen for both substances in the nanoencapsulated form, compared to their natural forms.

Vitamin D3 has been mentioned as an endogenous inhibitor of the hedgehog pathway, which has been seen to inhibit the cell growth of pancreatic adenocarcinoma in vitro and in vivo. Brüggemann et al. further investigated this effect [65]. Pancreatic carcinoma cells were successfully implanted, but no effects of vitamin D3 on tumor growth were detected. The authors concluded that vitamin D3 does not seem to have an effect in in vivo models since the same result was previously observed in a mouse model. Although some in vitro models showed an effect, the authors concluded that vitamin D3, as a monotherapy, is most likely not an effective therapeutic option for pancreatic cancer.

Furthermore, the effects of anticoagulants on pancreatic cancer were investigated in two studies. In 2014, Sudha et al. showed that both sulfated non-anticoagulant heparins (S-NACHs) and low molecular weight heparins (LMWHs) inhibited tumor growth and angiogenesis [66]. The combination of either substance with gemcitabine did not result in an increased reduction of tumor growth. In 2019, Featherby et al. studied the mechanisms by which LMWH and direct oral anticoagulants influenced tumor growth [67]. The VEGF-receptor-blocker Bevacizumab was sued as a comparison. While Tinzaparin did show a decrease in vessel density, no such effect was seen when direct oral anticoagulants were applied. No decrease in tumor growth was observed when different LMWHs were applied but a small reduction in size after treatment with Apixaban was seen.

Peulen et al. tested the effects of cyclooxygenase-2 (COX-2) and class I histone deacetylase (HDAC) inhibitors on pancreatic cancer cells [68]. While the treatment with just one of the substances markedly decreased the tumor growth, combined treatment stopped tumor growth. In 2021, Kumar et al. published a study in which they tested the effect of plasma-treated water (PTW) on pancreatic cancer cells [69]. The tumor growth was decreased after the application of PTW, and the analysis of the cytotoxic response revealed a reduction of glutathione peroxidase 4 (GPX4) and Glutathione (GSH). The expression of lipid peroxidation was increased.

Citrate represents an important player in tumor metabolism and particularly affects the interaction between tumor cells and the tumor microenvironment. Cancer cells endogenously synthesize citrate via the tricarboxylic acid cycle but also import extracellular citrate via the plasma membrane citrate transporter (pmCiC). As recently shown, the specific pmCiC inhibitor gluconate impairs cancer metabolism, proliferation, and metastases in vitro and in murine xenografts [70]. In 2021, Drexler et al. successfully engrafted L3.6pl cells (human PDAC cell line) and applied gluconate daily, starting on the second day after implantation which significantly reduced final tumor weight and volume [33].

However, not all substances can be tested on the CAM. In 2019, Skarbek tested whether arylboronate prodrugs of doxorubicin (DOX) were feasible chemotherapeutic agents for the treatment of PDAC [71]. Along with other methods, a decrease in tumor growth of PDAC cells was observed. After extraction of the tumor, the amount of free DOX was determined and led to the conclusion that well-developed tumors were able to convert the prodrug into the active substance.

**Table 3:** Overview of the aforementioned drug protocols that involved the CAM model as a drug testing platform for the assessment of pancreatic cancer.

Study	Drug	Dose/Duration	Application	Readout
[63]	Pomegranate fruit extract (flavonoids and polyphenols)	Single doses of 5–20 µg/CAM	Local application onto pancreatic tumor cells mixed with Matrigel	Pomegranate extract reduced tumor weight and angiogenesis
[64]	3,3′-diindolylmethane (DIM) Ellagic acid (EA) both DIM and EA	Single doses of 0.1–10 µg/CAM	Free form, or in nanoparticles onto pancreatic tumor cells mixed with Matrigel	Nanoencapsulation of DIM and EA together had a strong inhibiting effect on the tumor cell viability, angiogenesis, and tumor weight.
[65]	Vitamin D3	One dose/w for 2w of 0.01–100 µM/CAM	Local application onto pancreatic tumor cells mixed with Matrigel	Vitamin D3 did not show an effect in vivo but did reduce tumor cell growth in vitro
[66]	Tinzaparin (low molecular weight heparin, LMWH) Non-anticoagulant heparin (S-NACH) Gemcitabine (GEM) Tinzaparin and GEM S-NACH and GEM	Single dose of 1 µg/CAM	Local application onto pancreatic tumor cells mixed with Matrigel, not further specified	S-NACH and LMWH prohibited tumor growth and metastasis
[67]	Tinzaparin	Single doses of 1.25–5 IU/mL	Gelfoam absorbable gelatine pads soaked with tinzaparin were placed on pancreatic tumor cells mixed with Matrigel for 3 days	Tinzaparin at a concentration of 5 IU/mL significantly inhibited the angiogenesis of tumor cells on the CAM
[68]	Celecoxib, cyclooxygenase-2 (COX-2) inhibitor MS-275, class I histone deacetylase (HDAC) inhibitor Both, celecoxib and MS-275	Daily dose for 6 days of 8 µM/CAM of celecoxib 0.2 µM/CAM of MS-275 8 µM/CAM of celecoxib and 0.2 µM/CAM of MS-275	Local application directly onto pancreatic tumor cells mixed with Matrigel, not further specified	MS-275 decreased tumor growth, the combination stopped tumor growth; celecoxib did not affect tumor proliferation
[69]	Plasma treated water (PTW)	Single dose of 100 μL containing 10% PTW mixed with PBS	Local application directly into a sterile plastic ring containing tumor cells mixed with Matrigel	Reduction of tumor growth, PTW-derived oxidants induced ferroptotic cell death in pancreatic cancer cells
[33]	Gluconate, inhibitor of the plamsa membrane citrate tranporter (pmCiC)	Daily dose for 5 days of 4.5 mg/CAM	Local application directly onto pancreatic tumor cells mixed with Matrigel	pmCiC inhibition by gluconate reduced tumor growth
[71]	Arylboronate prodrugs of doxorubicin (DOX)	Single injection with 184 nmol, or twice a day for 2 days with 20 nmol/injection	Intratumoral injection into the pancreatic tumor formed with Matrigel	Arylboronate prodrugs inhibited the tumor growth. The prodrug was converted into DOX

### 3.4. Modifications

Several studies have investigated the overexpression of certain miRNAs in pancreatic cancer. Wei et al. studied the biological effect of miR-23-3p/ANXA2 Axis in PDAC [72] and were able to show that upregulating miR-23b-3p expression decreased tumor formation.

Costanza et al. analyzed whether the loss of transforming growth factor-beta-induced (TGFBI) expression in PDAC cells had any influence on tumor growth [73]. Transfected tumors were explanted on day 7 after implantation and the volumes were calculated. Tumor growth of PDAC cells with TGFBI depletion was significantly reduced and the amount of Ki67-positive protein was reduced as well, even though the effect was moderate.

A similar effect was seen after performing myoferlin silencing in PDAC cells [74]. Fahmy et al. noticed a significant decrease in tumor volume, as well as a drastic reduction of the blood vessel density within the tumor using SNA (sambucus nigra agglutinin) staining.

Dumartin et al. implanted PDAC cells after silencing the NTN1 gene in vitro and were able to show a strong decrease in tumor cell invasion into the CAM [75]. In addition, they detected human CA 19-9 in the blood of the chick embryo. Detection was possible on the first day after implantation and it increased over time.

In 2009, Schneiderhahn et al. were able to show that silencing of CD147 decreased the number of invasive tumors of MiaPaCa2 cells implanted onto the CAM assay by almost 50% [76]. Inducible short hairpin RNA-mediated CD147 was used for silencing.

Gharibi et al. described that integrin alpha 1 (ITGA1) is upregulated in pancreatic cancer [77] and modified the CAM model in order to use it as a metastasis assay to investigate the aforementioned upregulation. They grafted pancreatic cancer cells in the presence of exogenous collagen and TGFβ onto the CAM. After seven days, the tumor was excised and no effect of the depletion of ITGA1 on the average tumor weight was seen. This outcome contradicted the results of the in vitro experiments. However, a decrease in the development of metastases, mostly with regard to liver metastasis, was observed.

Agarwal et al. found phosphoribosylaminoimidazole-succinocarboxamide synthase (PAICS) to be overexpressed in PDAC and were able to correlate this with a poor prognosis [78]. For further evaluation, they implanted PAICS knockdown and PAICS stable pancreatic cancer cells onto the CAM. The results showed a decrease in the growth of the PAICS deficient tumors.

One of the major clinical problems when treating PDAC is its resistance to many of the current treatment regimens. Some studies indicate that the underlying cause could be attributed to defects in cell death programs [79]. In two studies, the X-linked inhibitor of apoptosis (XIAP) was analyzed as a possible therapeutic target for PDAC. In 2008, Vogler et al. showed that XIAP knockdown can enhance the tumor necrosis factor-related apoptosis-inducing ligand (TRAIL) induced antitumor activity, which resulted in suppression of tumor growth if XIAP knockdown cells were treated with TRAIL [80]. Yet, one of the mentioned approaches did not reduce tumor growth when it was applied solely. In 2009, the same group used small molecule XIAP inhibitors in combination with TRAIL to examine the tumor growth of pancreatic cancer cells on the CAM. The combination treatment was able to trigger apoptosis and reduce tumor growth.

### 3.5. Further Developments

One of the setbacks of the CAM model is the difficulty of determining an appropriate methodology for the objective evaluation of treatment effects such as tumor growth. This also applies to other possible read-outs such as blood flow or the optimal grafting period for different tumor types. Further developments of the CAM model are supposed to overcome these problems and optimize the research protocols.

In a study published in 2017, Rovithi et al. described the development of a bioluminescent CAM model [39]. They established four primary cell cultures from human PDAC from patients that received a pancreaticoduodenectomy, which they transduced with Flux expressing lentiviral vector. The cells were inoculated after establishing stability over several passages and proving that the bioluminescent signal correlated with the equivalent number of cells. They were able to show the growth of the bioluminescent signal over time, as well as the proportional increase of the tumor weight measured by caliper. Since only four models were established, the number of further possible experiments was limited. However, a pilot pharmacological study using either gemcitabine, crizotinib, or a combination of both was carried out. The results of this study show that treatment with one of the medications resulted in minor growth inhibition, while treatment with the combination therapy resulted in a significant decrease in mean Flux intensity.

PDAC is surrounded by a dense fibrotic stroma that contains pancreatic stellate cells which have been identified as one support factor for tumor growth in the nude mouse model [81]. Schneiderhahn et al. used the CAM model to further investigate the interactions between pancreatic stellate cells and PDAC cells [82]. For this purpose, they grafted PDAC cells either alone or in combination with pancreatic stellate cells onto the CAM. They observed tumor formation only in the presence of pancreatic stellate cells by PANC-1 cells. It must be noted that they used three different PDAC cell lines, and both MiaPaCa2 and SW850 cell lines did not form tumors either way. However, all cancer cell lines were able to induce tumorigenesis in the nude mice model and the combination with pancreatic stellate cells increased tumor weight in all three cell lines.

One of the problems that arise when resecting pancreatic cancer is the high rate of incomplete resections, which is partly due to its complicated anatomical location in conjunction with the very late diagnosis in most cases [83,84]. In a study published in 2012, Partecke et al. investigated the effect of tissue tolerable plasma (TTP) on human PDAC cell lines [85]. The term “plasma” is used in a physical sense in this context, which means that it relates to a fourth state of matter [86]. TTP is part of the group of non-thermal atmospheric plasmas. It forms different temperatures within itself, which is an attribute that can be used for medical purposes [87]. PDAC cells on the CAM were treated with TTP on day 12 of embryological development. After 48h, the tumors were explanted. The HE staining revealed changes in the cells in the upper three to five cell layers after treatment with TTP while no effect was observed in the lower layers. The authors suggest that the method might be a possible intraoperative application in the future in order to reduce microscopic tumor residue when resecting pancreatic cancer.

### 3.6. Personalized Medicine

Personalized medicine as a general term stands for a highly individualized approach toward research that is supposed to gain new insights by assessing different aspects that are specific for each individual patient in order to reduce side effects and develop more successful treatment strategies. However, these studies mostly aim at combining in vitro and in vivo treatment regimens which may include targeted gene therapy, antibodies or different chemotherapeutics. In this regard, pancreatic cancer cells derived from individual patients were grafted onto the CAM in two different studies.

In 2017 Ciolofan et al. grafted a cell suspension derived from a liver metastasis of pancreatic cancer patients onto the CAM [88]. If the tumors were visible two days after implantation, they were subsequently treated with either Bevacizumab, Rapamycin, or a combination of both three times every two days. On day 7 after implantation, the tumors were explanted, and immunohistochemical expression of CK7, CK19, and CK8/18 was performed in order to prove the pancreatic origin of the tumors. The treatment effects were evaluated by determining immunoexpression of CD34, podoplanin, PDGFA, and EGFR. The lowest expression was observed in the group that was treated with Rapamycin. The authors concluded that this does support the use of an mTOR inhibitor in the treatment of patients with PDAC liver metastases.

In 2014, Golan et al. grafted ascites-derived PDAC cells onto the CAM [89]. They used ascites from patients undergoing palliative paracenteses to establish primary cell cultures. Primary cell culture was successfully established in 92% of the obtained fluid samples from 36 different patients. Primary ascites-derived PDAC cells from eight different patients were transplanted onto 25 different CAMs. At the end of the growth period, larger masses were observed and successful engraftment was determined by HE staining and additional immunohistochemical staining of CK7. The results were very heterogenous with human cells only present in some embryos. Merely in one case, human DNA was detected in the embryonal liver tissue. There was a large heterogeneity regarding the behavior of the PDAC cells derived from different patients. The initial goal of the study was to use the model as a basis for personalized treatment plans in a short time frame. While the CAM model was not used for this purpose, the response of the individual cell cultures to different therapeutic agents was observed and a correlation between the in vitro results and the clinical outcome of the patients was seen.

## 4. Discussion

### 4.1. Choosing the Best Model

PDAC has been investigated in a large number of murine models, whereby the current focus appears to be on genetically engineered mouse models (GEMMs) (see Table 1) [90,91]. These models are very costly and time-consuming. They are not easily accessible and require specific knowledge as well as special facilities. In addition, there are increasing ethical concerns toward animal experiments not only within the public but also within the scientific community. The CAM model is currently not regarded as an animal model in many countries, and therefore not subjected to the same rules and regulations which increases the accessibility and makes the implementation of studies a lot easier for researchers.

### 4.2. The CAM Model: Advantages and Disadvantages

Some of the main advantages of the CAM model are versatility, simplicity, and low cost. Yet, careful monitoring during the experimental use is still necessary because of its high susceptibility to environmental factors, such as pH, osmolarity, humidity, and oxygen availability [92]. Also, the eggs used for this methodology have to be free of pathogens such as fungi and contamination is a common problem [93]. There are no standardized protocols, which makes it very difficult to compare studies using the CAM model because changes in CAM preparation, as well as different ways of engrafting (such as using a silicon ring or matrix gel), can result in multiple outcomes. One of the problems of the CAM model is the distinction between neovascularization and rearrangement of preexisting vessels. Therefore, the preexisting blood vessels must be carefully monitored from the start of embryonic development. Another problem is caused by an unspecific inflammatory reaction through cell implantation or shell fragments that fall onto the CAM while opening the egg. The inflammatory response might lead to angiogenesis [54], however, the inflammatory response of the chick embryo is usually limited, since its immune system is not fully developed until about day 14 of embryonic development. This also means that substances which influence the immune response are difficult to test in the CAM model [94].

The CAM model has both advantages and disadvantages in comparison to other in vivo tumor models. Büchler et al. tried to induce hypoxia by applying cycloheximide [58] which appeared to be lethal for most of the chicken embryos. Furthermore, the experimental model depends on the survival of the chick embryo which limits the substances as well as the concentrations that can be examined using this model. To find the correct dose, the upper limit (lethal dose) for the embryo has to be determined first [71]. Therefore, substances that might require a higher serum level for sufficient pharmacodynamic action cannot be tested using the CAM model. Also, the chicken embryos must be treated carefully and monitored consistently throughout the entire experiment. Additionally, some substances might not be compatible with avian species at all due to a number of different reasons such as a different receptor composition for example. The tested substances can either be applied topically or can be injected into one of the blood vessels using a microcapillary syringe. Oral application, which is performed in mouse models, is not possible. These methods do not reflect the possible systemic drug turnover or modifications that might occur in an immunocompetent human. Although some studies showed that the transformation of a prodrug into an effective drug by pancreatic tumors on the CAM is possible, these processes must be carefully evaluated for every single substance tested [71].

When using the CAM model as a drug testing platform, it is of utmost importance to determine the correct concentrations that are sufficient for human treatment. However, most chemotherapeutics are applied intravenously, which makes it difficult to determine what dosages may be effective from merely intratumoral injection or local treatment on the CAM. Regarding this topic, Kue et al. found that the median lethal dose and the median survival dose of several FDA-approved chemotherapeutics injected into CAM vessels appeared to moderately correlate with intravenous and intraperitoneal application doses for rodents [95]. This may indicate that one can adjust the anticancer drug screening doses for preclinical trials using the CAM model. Even though intravenous injection may appear more challenging, it could possibly yield more information on the pharmacodynamics of the tested substance.

### 4.3. Technical Aspects

There are several ways in which tumor growth is currently measured. One is to explant and weigh the tumor, which poses the disadvantage that it does not allow for repeated measurements. This is also the case when total tumor cell counts are performed [96,97,98]. Another possibility is represented by calculating the tumor mass using photographs or real-time imaging software. However, these are only estimations and are very susceptible to inter-researcher bias. Other approaches aimed at objectifying the results by using specific software for the analysis, but the results remain estimations. One technique described by Rovithi et al. using bioluminescence might complement these methods and would allow for repeated measurements over time [39]. Regarding angiogenesis, tumor hemoglobin content has been used as an index for the vascularity of the tumor [99]. The Hb concentration can be compared to a standard curve to evaluate the results. Other ways of evaluating experimental results such as morphological responses are very difficult to objectify. Not only does inter-rater bias have to be taken into consideration, but so do the many variables that can confound the results of the experiment. For example, the age of the embryo, non-specific inflammation, and the effect an applied substance might have on the CAM and the chicken embryo itself [100].

The CAM model has the potential to be used as a platform for personalized medicine. If specimens from tumor biopsies can be efficiently grafted onto the CAM, this could lead to new insights into the characteristics of individual tumor samples and it might even be a platform for individual drug testing. The high level of heterogeneity of PDAC must be considered though because the risk of a sampling error could be high.

## 5. Conclusions

Pancreatic cancer cells have been grafted onto the CAM model in a variety of studies including angiogenesis, drug testing, and personalized medicine. While the CAM model can be a useful tool to gain a more profound understanding of the complex nature and heterogeneity of PDAC, further studies with mammalian models will be necessary once a potential substance has been identified using the CAM model.

## Figures and Tables

**Table 2:** Summary of the aforementioned studies that assessed the use of the CAM model for the assessment of tumor-induced angiogenesis in pancreatic cancer.

Study	Readout	Results
[58]	Hypoxia-induced de novo transcription of uPAR mRNA in pancreatic cancer cell lines	Tumor-induced angioinvasion of human pancreatic cancer cells in vitro and in vivo may depend on hypoxia
[59]	Depletion of PKD2 in the endothelium in sprouting assays and tumor xenografts inhibited tumor-induced angiogenesis of pancreatic cancer cells	PKD2 controls hypoxia-induced VEGF-A expression, secretion, and blood vessel formation of pancreatic and gastric tumors
[60]	The sst2-dependent upregulation of TSP-1 slowed down tumor cell-induced blood vessel formation by encapsulating VEGF and inactivated the endothelial effects of VEGFR2	TSP-1 and sst2 function as tumor suppressors and could suppress the proliferation of pancreatic cancer
[61]	Inhibition of EGFL7 expression restricted microvessel formation of pancreatic carcinoma by downregulation of VEGF and Ang-2	EGFL7 is a possible marker for prognosis and perhaps a therapeutic target of pancreatic carcinoma
[62]	Prognostic values and expression of Ezrin on Akt/mTOR pathway and YAP expression in pancreatic cancer and healthy pancreas tissue was assessed in different assays	Ezrin and YAP are overexpressed in pancreatic cancer and correspond with a poor prognosis
